# Predicting Unobserved Phenotypes for Complex Traits from Whole-Genome SNP Data

**DOI:** 10.1371/journal.pgen.1000231

**Published:** 2008-10-24

**Authors:** Sang Hong Lee, Julius H. J. van der Werf, Ben J. Hayes, Michael E. Goddard, Peter M. Visscher

**Affiliations:** 1 School of Environmental and Rural Science, University of New England, Armidale, New South Wales, Australia; 2 National Institute of Animal Science, Rural Development Administration, Cheon An, Korea; 3 Department of Primary Industry, Victoria, Australia; 4 Faculty of Land and Food Resources, University of Melbourne, Melbourne, Australia; 5 Queensland Institute of Medical Research, Brisbane, Australia; University of Wisconsin, Madison, United States of America

## Abstract

Genome-wide association studies (GWAS) for quantitative traits and disease in humans and other species have shown that there are many loci that contribute to the observed resemblance between relatives. GWAS to date have mostly focussed on discovery of genes or regulatory regions habouring causative polymorphisms, using single SNP analyses and setting stringent type-I error rates. Genome-wide marker data can also be used to predict genetic values and therefore predict phenotypes. Here, we propose a Bayesian method that utilises all marker data simultaneously to predict phenotypes. We apply the method to three traits: coat colour, %CD8 cells, and mean cell haemoglobin, measured in a heterogeneous stock mouse population. We find that a model that contains both additive and dominance effects, estimated from genome-wide marker data, is successful in predicting unobserved phenotypes and is significantly better than a prediction based upon the phenotypes of close relatives. Correlations between predicted and actual phenotypes were in the range of 0.4 to 0.9 when half of the number of families was used to estimate effects and the other half for prediction. Posterior probabilities of SNPs being associated with coat colour were high for regions that are known to contain loci for this trait. The prediction of phenotypes using large samples, high-density SNP data, and appropriate statistical methodology is feasible and can be applied in human medicine, forensics, or artificial selection programs.

## Author Summary

Results from recent genome-wide association studies indicate that for most complex traits, there are many loci that contribute to variation in observed phenotype and that the effect of a single variant (single nucleotide polymorphism, SNP) on a phenotype is small. Here, we propose a method that combines the effects of multiple SNPs to make a prediction of a phenotype that has not been observed. We apply the method to data on mice, using phenotypic and genomic data from some individuals to predict phenotypes in other, either related or unrelated, individuals. We find that correlations between predicted and actual phenotypes are in the range of 0.4 to 0.9. The method also shows that the SNPs used in the prediction appear in regions that are known to contain genes associated with the traits studied. The prediction of unobserved phenotypes from high-density SNP data and appropriate statistical methodology is feasible and can be applied in human medicine, forensics, or artificial breeding programs.

## Introduction

Results from linkage analyses and, more recently, genome-wide association studies (GWAS) imply that a large number of loci underlie the genetic architecture of complex traits [1]–[15]. GWAS are usually multi-staged, have mostly focused on gene discovery and typically set very stringent type-I error rates in the first stage to avoid false positives. Analysis is most frequently performed one SNP at a time. Consequently, these studies may not properly capture all of the genetic variation that is present in the samples, The initial wave of GWAS has found many genetic variants that are robustly associated with disease or quantitative traits, but these variants typically explain only a small fraction of the genetic variance, and so the utility of predictions made using this information can be limited.

An alternative to gene discovery is to focus on the prediction of phenotypes using all genotypic (SNP) information across the whole genome simultaneously. The prediction of phenotypes is useful in a range of fields, from artificial selection programs [16] to risk prediction in human medicine [17] and forensics. To predict phenotypes, identification or genotyping of causal variants is not necessary, as long as there are variants genotyped that are in linkage disequilibrium (LD) with the causal variants [16],[17].

To predict phenotypes from genomic data, the relationship between genome-wide marker data and phenotypes needs to be modeled. The single SNP regression approach that is often applied in conjunction with stringent thresholds would be expected to inaccurately estimate the proportion of variance that can be explained from genotypic data. Instead, model selection approaches are required to find the set of SNPs that best explains and predicts variation in phenotype. Such approaches have already been proposed for mapping multiple quantitative trait loci (QTL) [18]–[23] and recently a method was suggested for the simultaneous analysis of all SNPs in a GWAS [24].

In this study, we use statistical modeling to fit multiple SNP effects from a GWAS and derive the best model with a Bayesian model selection approach termed *Reversible Jump Markov Chain Monte Carlo* (RJMCMC) [25]. We predict unobserved phenotypes for individuals based on genome-wide SNP data only, family information (without genetic data) only, or on a combination of the two.

## Methods

### Data

Publicly available data including pedigree, genotypic and phenotypic information on heterogeneous stock mice were used ([26]; http://gscan.well.ox.ac.uk/). The total number of animals was 2,296 from 85 unrelated families. The available pedigree spanned four generations, generating complex relationships. In the last generation, there were 172 full sib families with an average size of ∼11 (SD ∼8). Genotypes were available for 12,112 SNPs on most animals in the pedigree, and we used the 11,730 SNPs on the autosomal chromosomes. Phenotypes were already adjusted for the environmental fixed effects, e.g. sex, age, year and season [26],[27]. We chose three phenotypes, coat colour as a complex trait with a number of known causal loci (estimated *h^2^*≈0.72), and percentage of CD8^+^ cells (%CD8) as a quantitative trait having high heritability (estimated *h^2^*≈0.99), and mean cellular haemoglobin (MCH) as a quantitative trait having moderate heritability (estimated *h^2^*≈0.55). Coat colour, as used here, is a measure of the darkness of the coat from white to black. For more detail about the data, see [26],[27].

### Models

We fitted a range of linear mixed models, with multiple SNPs as fixed effects and, in some models, a polygenic effect to account for additive genetic effects not detected by the SNPs. These polygenic effects are estimated from the pedigree. The effect of a SNP genotype on the phenotype was modeled either by fitting the additive term of one of the alleles or by fitting both additive and dominance terms.

### Additive Genetic Model (Model A)

In the additive genetic model, phenotypic observations are a linear function of fixed effects, a polygenic term representing the sum of unidentified additive genetic effects, the additive effects due to SNPs associated with QTL and residuals. The linear model can be written as,
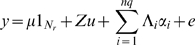
(1)where *y* is a vector of length *N_r_*, with single trait phenotypes for all animals corrected for fixed environmental effects (*N_r_* = no. observations in Table 1), *nq* is the number of SNPs associated with the QTL involved in phenotypic expression, *μ* is the overall mean, 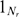 is a vector of *N_r_* ones, *u* is a vector of *N* random polygenic effects for *N* animals (*N* = 2296), *α_i_* is the fixed effect of the i^th^ SNP and *e* is a vector of residuals. *Z* is an incidence matrix for the random polygenic effects relating observations to individual animals, with dimensions *N_r_*×*N*. Note that N>N_r_ as some animals have a polygenic effect estimated based upon phenotypic information from relatives without having a phenotypic observation themselves. Λ*_i_* is a column vector of length *N_r_* having coefficients 0, 1 or 2 representing indicator variables of the genotype for each animal at the i^th^ SNP. The variance structure of phenotypic observations is written as 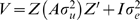, where *A* is the numerator relationship matrix, *I* is a identity matrix, 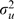 is polygenic additive genetic variance and 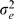 is error variance.

**Table 1:** The number of observations (and SD^a^) in the entire data set and the test and prediction sets.

Trait	Total no. observations	Strategy	No. observations	
			Estimation set	Prediction set
coat colour	1940	intra-family	975 (12)	965 (12)
		inter-family	993 (237)	947 (237)
%CD8	1410	intra-family	714 (14)	696 (14)
		inter-family	719 (177)	691 (177)
MCH	1580	intra-family	797 (11)	783 (11)
		inter-family	800 (200)	780 (200)

a Standard deviation over 10 replicates.

### Additive and Dominance Genetic Model (Model AD)

In the model containing additive and dominance effects, all terms are the same as the additive genetic model except that dominance effects due to SNPs are added. The model is written as,
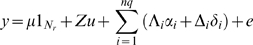
(2)where *δ_i_* is the dominance effect of the i^th^ SNP and *Δ_i_* is a column vector having coefficients that are 1 for a heterozygous genotype and 0 for a homozygous genotype at the i^th^ SNP.

### Estimation of Effects and Model Selection

#### Reversible Jump MCMC to Simultaneously Consider the Whole Genome

The number of QTL (*nq*) across the whole genome involved in phenotypic expression and the position of each QTL (*ρ_i_*, *i* = 1−*nq*) were sampled, and maximum likelihood (ML) estimates for *u*, *α* and *δ* (Θ) are obtained in every MCMC round. In each MCMC round, unknown phenotypes for individuals are predicted based on estimates of *u*, *α* and *δ*, and predicted phenotypes are finally averaged over all MCMC rounds. The probability of the sampled parameters given observed phenotypes is
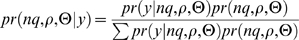
(3)where *pr*(*y*|*nq*, *ρ*, Θ) is the likelihood of the observed phenotypes given the sampled variables, *pr*(*nq*, *ρ*, Θ) is the joint prior probability of the variables, and the denominator is summed over the probabilities of all possible parameter states. If the parameter states are many, a MCMC method can be an efficient tool to obtain the posterior distribution for the parameters. When varying the number of QTL in the model, the model dimension varies. A Metropolis-Hastings sampler cannot properly infer the correct distribution unless the model dimension is fixed. However, a reversible jump MCMC [25] can communicate across all possible states of different dimensions according to the proper acceptance ratio and give the correct posterior distribution [28]. We give more details on the RJMCMC procedure in the online Supporting Information (text S1).

The polygenic heritability used in models **A** and **AD** was fixed as 0.72, 0.99 and 0.55, for coat colour, CD8% and MCH, respectively. We adjusted the phenotypes for the estimated polygenic effects using best linear unbiased prediction (BLUP, [29]) and then proceeded with modeling the SNPs. This two-stage procedure was done in every MCMC round because of computational efficiency. The heritabilities used were estimates of the total additive variance whereas in our model they should have been only the additive variance not explained by the SNPs in the model. However, using a heritability that is too high is conservative in that it reduces the likelihood that a SNP will be included in the model. Using a constant heritability, rather than re-estimating it in every round of MCMC, saved computer time which was important in carrying out multiple replicates of the analysis to estimate the accuracy of predicted phenotypes. The length of the MCMC chains was 10,000, in addition to an initial 1000 iterations of burn-in.

#### Single SNP Analyses

To perform a comparison with a ‘standard’ GWAS analysis, multiple SNP models were compared to single SNP analyses. In these analyses, the model **AD** (2) was used but fitting only a single SNP at a time. To mimic a typical GWAS analysis, we used linear regression of the phenotype on indicator variables for additive and dominance effects at a single SNP, analysed the data by maximum likelihood, calculated a likelihood-ratio test statistic for each SNP and selected the best set of SNPs according to a pre-defined threshold for the test statistic. We used (genome-wide) thresholds of 10.83, 15.14, 21.14 and 24.24, corresponding to nominal significance levels of 0.0045, 0.00052, 0.000026 and 0.0000055, respectively, assuming that the test statistic is distributed as a χ^2^ with 2 degrees of freedom. However, due to linkage disequilibrium there were many significant SNPs within a small region, and only the SNP with the largest test statistic within a 4 cM region was chosen.

### Predicting Unobserved Phenotypes

We predicted phenotypes of individuals by using information on relatives and/or the estimated effects of their SNP genotypes. Prediction of phenotypes was based on BLUP of polygenic values [29] using pedigree and phenotype information only (i.e. (1) with the third term omitted), or on additional genomic information where the prediction model was based on additive effects only (model **A**) or on both additive and dominance effects (model **AD**). Both **A** and **AD** models were fitted with and without the effects of additional polygenic factors from pedigree relationships. For the single SNP analyses, the prediction was performed from a multiple regression analysis using those SNPs that were selected previously from the single SNP analyses. As for the **AD** model, the single SNP analyses also fitted polygenic effects, plus the additive and dominance effect of the SNP.

To assess how well we predicted unobserved phenotypes, we used one part of the data for estimation and the remaining part for prediction and validation. Approximately half of the phenotypic data for each trait were randomly selected. Using only half of the phenotypes and all genotypes, the other half of the phenotypes (i.e. future, unobserved, phenotypes) were predicted with the proposed genetic model using whole genome SNP data. We tested how well we could predict phenotypes from genetic data in two ways. The first prediction was within families, using phenotypic data from approximately half of the animals in each full sib family to predict the phenotypes of the other half of the animals (intra-family comparison). The second prediction was across families, using phenotypic data from approximately half of the 85 unrelated families to predict the phenotypes of the animals in the other half of the families (inter-family comparison). The latter prediction could also be used for data sets that lack pedigree information. When fitting the pedigree only, i.e. not using any marker data, there is no ability to predict the phenotypes of animals in other, unrelated families, so the accuracy of the inter-family prediction is zero.

For each comparison, we correlated the predicted genotype of an animal in the prediction set with its phenotype (which was not used in the estimation phase). We term the correlation between predicted phenotypes and actual phenotypes as the accuracy of prediction. To gauge the precision with which this correlation is estimated we performed 10 replicates. For each replicate, the estimation and prediction sets were sampled and analyzed.

In addition to performing the model selection procedure and prediction from the entire autosomal SNP genotype set, we also investigated how well genotypic data from a single chromosome could predict phenotypes. For individual chromosome analyses, the **AD** model was used for the inter-family prediction with a single replicate per trait.

## Results

### Unobserved Phenotypes Can Be Predicted from Genome-Wide SNP Data

The total number of original phenotypes, the number of phenotypes used in the estimation analysis and the number of phenotypes to be predicted but not used in the estimation step are shown in Table 1. For the prediction set, on average approximately 700 (%CD8) to 950 (coat colour) observations were used.

Table 2 shows the correlation between true and estimated phenotypes of the three different traits when using the intra- or inter-family prediction. It shows that the use of genomic information substantially increases the accuracy of predicting unobserved phenotypes, compared to BLUP (fitting only the pedigree), and a substantial accuracy was achieved even with inter-family prediction, where genomic and phenotypes data in some families was used to predict phenotypes in other families. The accuracy of prediction is highest with intra-family prediction when using genomic information and phenotypic information from relatives to predict an individual phenotype. For example, for %CD8 and an additive model of gene action and fitting the pedigree, the correlation between predicted and observed phenotype is 0.71 whereas it is 0.64 when using only pedigree information.

**Table 2:** Correlation (SD^a^) of actual and predicted phenotypes and their standard deviations^a^.

Model	Intra-family wise			Inter-family wise		
	Coat colour	%CD8	MCH	Coat colour	%CD8	MCH
BLUP (Ignoring genotypic data)	0.54 (0.02)	0.64 (0.02)	0.41 (0.01)	0.00	0.00	0.00
Fitting genotypic data and pedigree						
Model A	0.72 (0.02)	0.71 (0.02)	0.52 (0.02)	0.58 (0.06)	0.50 (0.05)	0.35 (0.07)
Model AD	0.89 (0.03)	0.73 (0.02)	0.55 (0.02)	0.87 (0.05)	0.58 (0.05)	0.36 (0.09)
Fitting genotypic data and ignoring pedigree						
Model A	0.65 (0.02)	0.65 (0.02)	0.46 (0.04)	0.54 (0.06)	0.51 (0.05)	0.33 (0.06)
Model AD	0.85 (0.04)	0.69 (0.02)	0.50 (0.04)	0.81 (0.08)	0.56 (0.06)	0.33 (0.09)

a Standard deviation over 10 replicates.

The accuracies of prediction with the model **AD** are generally greater than those with model **A** for intra- and inter-family prediction. The difference between the accuracies with and without considering dominance varies across the traits. For coat colour, the accuracy of prediction substantially increases in both intra-family (0.72 to 0.89) and inter-family (0.58 to 0.87) prediction. For %CD8, the accuracy increases slightly for the intra-family prediction (0.71 to 0.73). The increase due to inclusion of dominance is larger for the inter-family prediction (0.50 to 0.58). For MCH, the accuracy slightly increases for both intra- and inter-family prediction. These results are consistent with a substantial amount of dominance variance for coat colour, some dominance variance for %CD8 and little dominance variance for MCH.

When omitting polygenic terms in the genetic model and using whole genome marker information only, the correlations between predicted and actual phenotypes are generally decreased for the intra-family prediction, and practically unchanged in inter-family prediction except for coat colour (Table 2). The bottom two rows of Table 2 for the inter-family prediction show that phenotypes can be predicted from marker data and phenotypes observed in ‘unrelated’ families. For coat colour and the **AD** model, the prediction is very good (correlation of 0.81).

### Prediction of Genetic Factors

The precision with which phenotypes can be predicted from genetic data is, of course, limited by how much of the variation between individuals is due to genetic factors. Prediction of unobserved phenotypes from genetic data will never be accurate for traits with a low heritability, even if the prediction of the genetic effect is 100% accurate. To quantify how much of the variation between individuals due to genetic effects we detected, we scaled the accuracy of predicting phenotypes by *h*, the square root of the heritability. This parameter represents the correlation between additive genetic value and phenotype, and is a key parameter in artificial selection programs [30]. The scaled accuracy is an estimate of the precision with which additive genetic values are predicted. When using an additive genetic model and whole genome information (Model **A**), this estimated correlation between predicted and inferred genetic values for the intra-family prediction was 0.84, 0.71 and 0.71 for coat colour, %CD8 and MCH, respectively, and 0.68, 0.50 and 0.47 for the inter-family prediction (Table 3). When using an additive and dominance genetic model and whole genome information (Model **AD**), the estimated correlation between predicted and inferred additive genetic values for the intra-family prediction was 1.05, 0.73 and 0.75 for coat colour, %CD8 and MCH, respectively, and 1.02, 0.59 and 0.48 for the inter-family prediction (Table 3). Therefore a large proportion of existing genetic variation was detected and exploited by our application. It should be noted that the values for model **AD** should be scaled by the square root of the broad-sense heritability which was, however, unknown. Instead, we scaled the values for the **AD** model by narrow-sense heritability, which may result in an overestimation of accuracy depending on the amount of dominance variance.

**Table 3:** Correlation (SD^a^) between predicted and inferred additive genetic values.

	Intra-family wise			Inter-family wise		
	Coat colour	%CD8	MCH	Coat colour	%CD8	MCH
BLUP	0.63 (0.03)	0.64 (0.02)	0.55 (0.02)	0.00	0.00	0.00
Model A	0.84 (0.02)	0.71 (0.02)	0.71 (0.03)	0.68 (0.07)	0.50 (0.05)	0.47 (0.09)
Model AD	1.05 (0.04)	0.73 (0.02)	0.75 (0.03)	1.02 (0.06)	0.59 (0.05)	0.48 (0.12)

a Standard deviation over 10 replicates.

### Advantage of a Whole-Genome Approach

**Figure 1:**
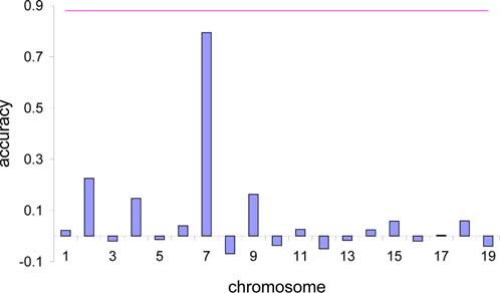
Correlation between predicted and actual phenotype for coat colour. Results from the additive and dominance genetic model and inter-family prediction when using each chromosome at a time (vertical bars), and when using whole genome information (horizontal line, 0.88).

**Figure 2:**
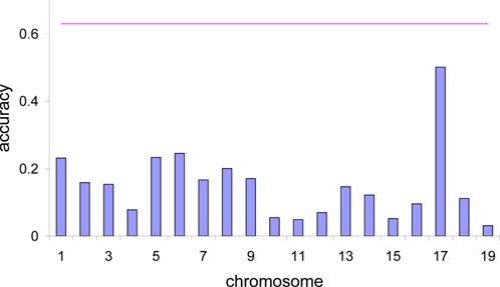
Correlation between predicted and actual phenotype for %CD8. Results from the additive and dominance genetic model and inter-family prediction when using each chromosome at a time (vertical bars), and when using whole genome information (horizontal line, 0.63).

**Figure 3:**
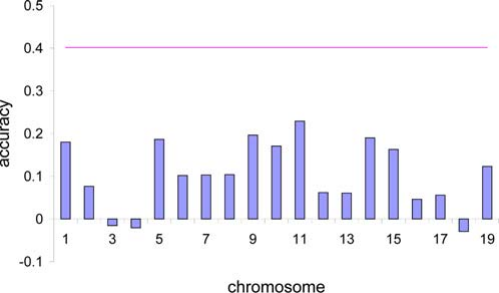
Correlation between predicted and actual phenotype for MCH. Results from the additive and dominance genetic model and inter-family prediction when using each chromosome at a time (vertical bars), and when using whole genome information (horizontal line, 0.4).

Figures 1, 2 and 3 show that the accuracy of prediction is higher when considering whole genome information compared with using information from one chromosome at a time. Even with coat colour, a single gene or a single chromosome does not determine all variation in phenotypic expression (Figure 1). Although the accuracy of prediction when considering chromosome 7 alone is high (0.79), the accuracy can be improved when using whole genome information (0.88). With %CD8 (Figure 2), the accuracy of prediction obtained by considering each chromosome at a time ranges from 0.05 to 0.50, implying that most chromosomes contribute to variation in this complex phenotype. When considering the entire genome simultaneously, the accuracy of prediction increases to 0.63. With MCH, the accuracy obtained from individual chromosomes varies up to 0.23 (Figure 3). However, again the accuracy of prediction is highest (0.40) when using whole genome information. The estimated negative correlations between actual phenotypes and predictions based upon a single chromosome (e.g., Figure 1) is most likely due to sampling error. Chromosomal analyses were done for a single replicate.

### Location of Trait Loci from Whole-Genome Estimation

**Figure 4:**
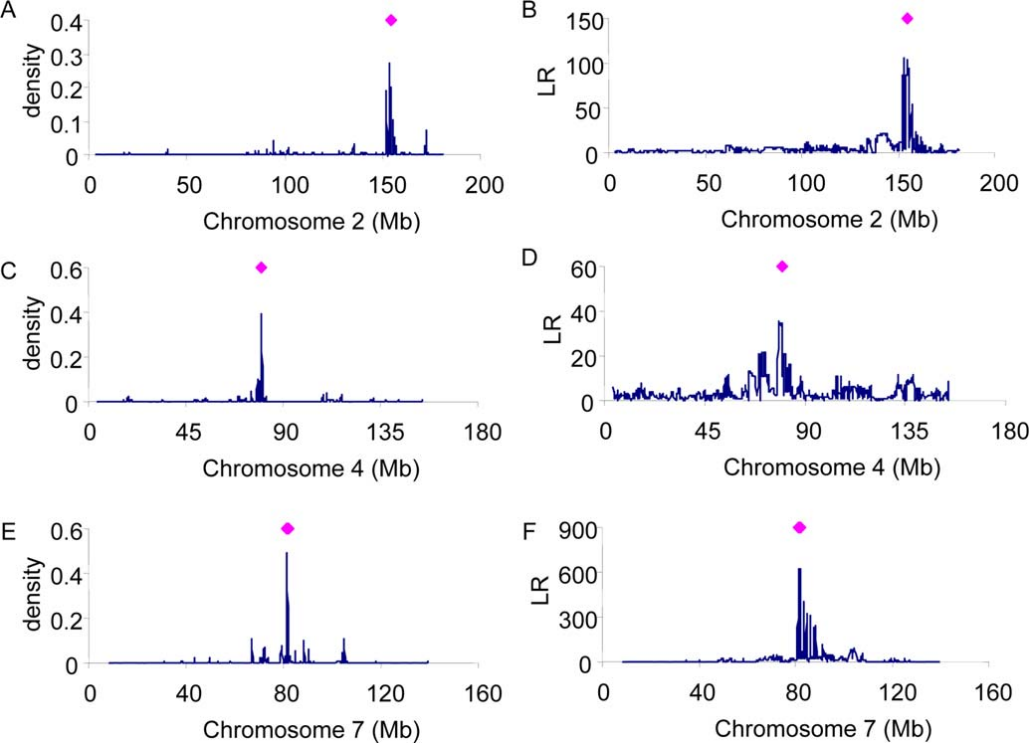
Posterior density of association of SNPs for coat colour using the whole-genome approach (A, C, E) or Likelihood Ratio of single SNP regression (B, D, F). For comparison, the positions of known genes for coat colour are shown (diamonds).

The whole genome approach based on fitting multiple SNPs and using RJMCMC for model selection provides a posterior density of each SNP being associated with the phenotype. Therefore, the positions of trait loci can be estimated (e.g. Figure 4A, C and E). For comparison, the method using regression on single SNPs that considers one position at a time was used. This method yields a likelihood ratio (LR) for each SNP which was plotted against genomic position (Figure 4B, D and F). Averages of the posterior QTL density or LR from the 10 replicates are shown for the inter-family prediction.

For coat colour, high posterior densities are shown for the regions around ∼159 Mb on chromosome 2, ∼80 Mb on chromosome 4 and ∼80 Mb on chromosome 7 (Figure 4 A, C and E). These regions agree very well with the positions of a number of known genes for variation in coat colour [31] (diamonds in Figure 4). Specifically, the non-agouti gene is at 154 Mb on chromosome 2, tyrosinase-related protein is at 79 Mb on chromosome 4, and the tyrosinase and Rab38 genes are at 81 Mb and 82 Mb, respectively, on chromosome 7. The LR profiles from the single SNP method are similar to that from the multiple SNP method (Figure 4B, D and F). However, correlated estimates due to linkage disequilibrium between the causal genes and multiple SNPs cause a broad confidence interval when using the single SNP method.

**Figure 5:**
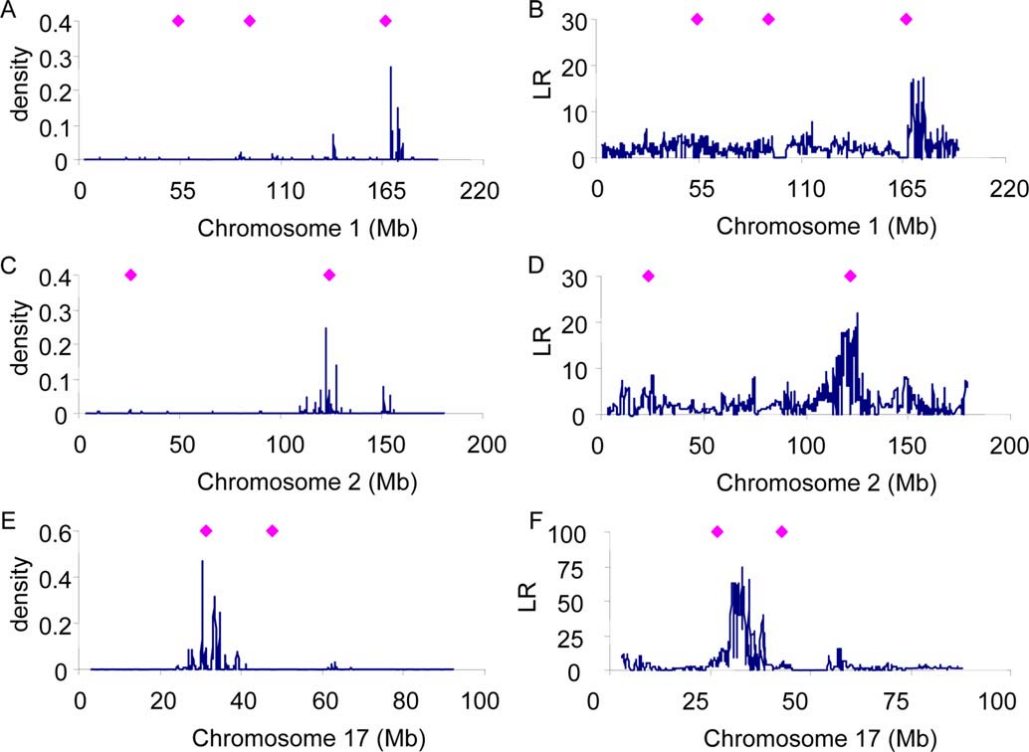
Posterior density of association of SNPs for %CD8 using the whole genome approach (A, C, E) or Likelihood Ratio of single SNP regression (B, D, F). For comparison, the positions of known genes for %CD8 are shown (diamonds).

For %CD8, high posterior densities are shown for the regions around ∼170 Mb on chromosome 1, ∼125 Mb on chromosome 2 and ∼30 Mb on chromosome 17 (Figure 4A, C and E). Some of these estimated positions agree with putative QTL region previously reported by [26] (also see http://gscan.well.ox.ac.uk/) (diamonds in Figure 5). The LR pattern from the single SNP method is similar to that from the multiple SNP method (Figure 5B, D and F), but again the mapping resolution is lower.

**Figure 6:**
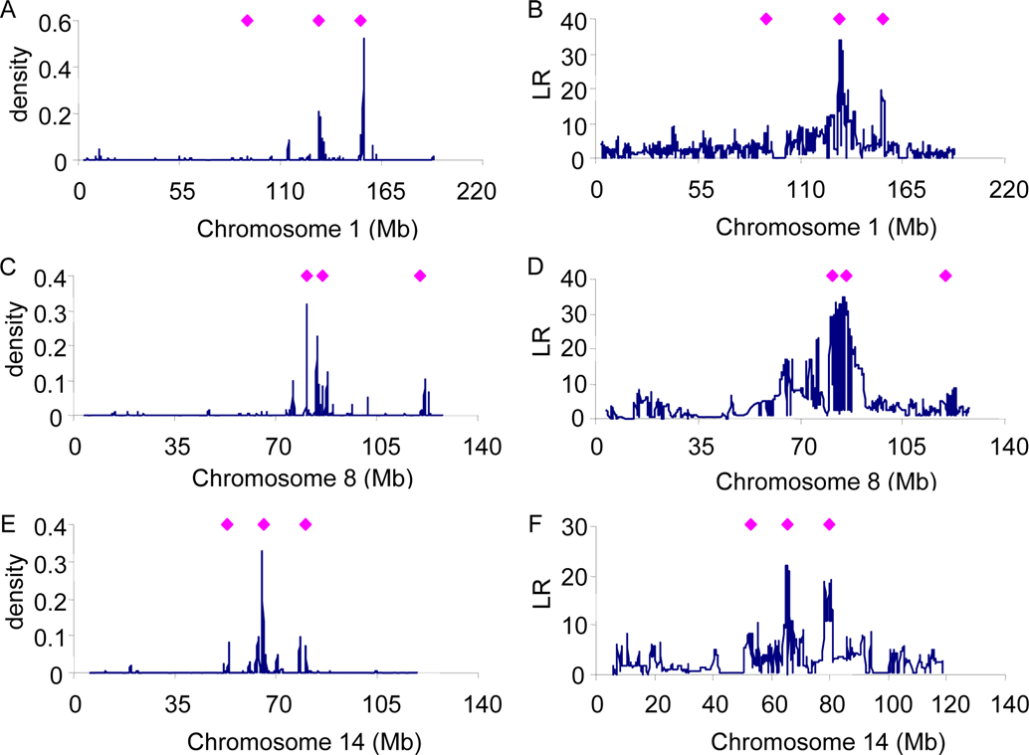
Posterior density of association of SNPs for MCH using the whole genome approach (A, C, E) or Likelihood Ratio of single SNP regression (B, D, F). For comparison, the positions of known genes for MCH are shown (diamonds).

For MCH, high posterior densities are observed for the region near ∼155 Mb on chromosome 1, ∼82 Mb on chromosome 8 and ∼65 Mb on chromosome 14 (Figure 6A, C and E). Estimated positions agree well with putative QTL region previously reported [26] (diamonds in Figure 6). As with the other traits, the single SNP method has lower map resolution.

### Convergence of Parameter Estimates

**Figure 7:**
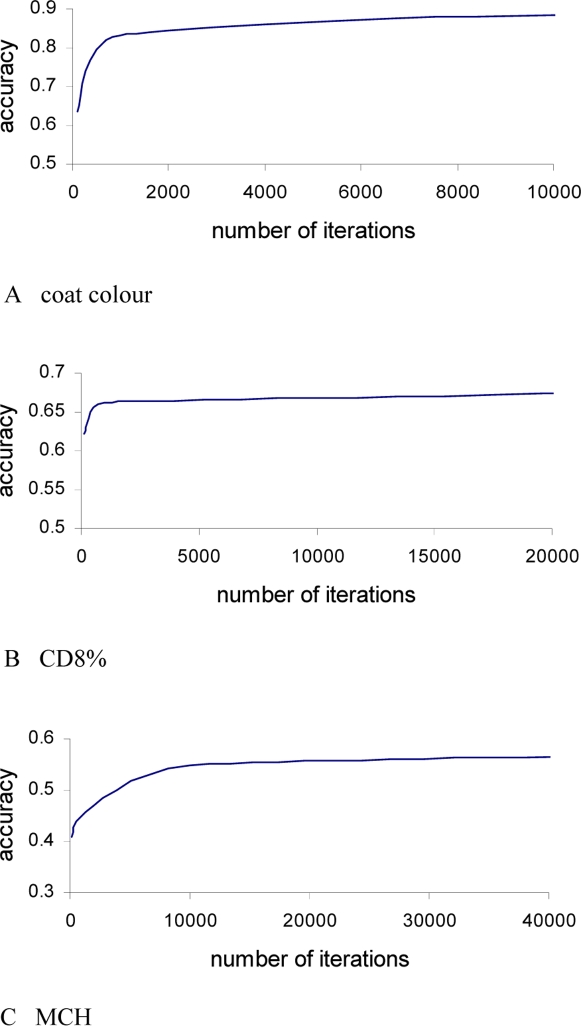
Convergence diagnostics for the values of the accuracy of predicting unobserved phenotypes.

Convergence of the parameter estimates was diagnosed from the pattern of the accuracy values after 100, 1000, 10000 and 100000 iterations when using intra-family prediction for a single replicate. The burn-in period was 10% of the total number of iterations. Figure 7 shows that the accuracy rapidly increases in early iteration rounds, and generally becomes a stable value after 10,000 iterations. A similar pattern was observed in the inter-family prediction, i.e. the accuracy reached a stable value after ∼10,000 iterations (result not shown), indicating that only a moderate number of iterations are required to achieve the accuracies of predicted phenotype shown in the results. The pattern of convergence of the estimated parameters (e.g. variances) was similar to that of the accuracy (result not shown), which was expected because accuracy was closely related to the estimated parameters. In this study, we used only a single starting value in order to save computing time due to many different situations to be tested with many analyses. However, for a single intensive analysis, it is always desirable to use multiple starting values to make sure that estimates reach apparent convergence.

## Discussion

We have proposed a method to simultaneously analyse whole genome SNP data for association with phenotypes, applied this method to three traits measured in a heterogeneous mouse stock and successfully predicted unobserved phenotypes. The precision of the prediction of unobserved phenotypes depends on the actual genetic architecture of the traits (heritability, number of genes, distribution of effect sizes and mode of gene action), the marker density and experimental sample size. For the qualitative trait (coat colour) and the highly heritable quantitative trait (%CD8), the accuracies of predicting phenotypes were high, even when using genomic information from unrelated families in the same population. This is a valuable result with important applications in medicine, agriculture and forensics.

Reversible jump theory is well established for solving model selection problem [20], [25], [32]–[34]. We found that RJMCMC in genomic selection was computationally efficient and gave reliable estimates. For the data set on mice (∼2200 individuals and ∼10,000 SNP), it took ∼15 minutes with a single CPU (∼2 GHz), which compares favourably to a number of other computing strategies on the same data set [35]. Assuming that computing time increases linearly with the number of individuals and markers, the method would run within one week even if the data set was large (e.g. 10,000 individuals with 1,000,000 SNPs). More time may be required to adequately monitor convergence, however parallel computing strategies would be useful here, e.g. [36]. Therefore, the methods described in this study can scale up to much larger data sets.

There are several approaches for whole genome association studies such as Bayesian random effect approaches [37], ridge regression or shrinkage estimators [38],[39]. However, most of these approaches are computationally intensive (as reported by [37]–[39], and some statistical properties are ill-defined (as discussed in [38]). Data sets used in those studies [37]–[39] were much smaller than what we have used here. Nevertheless, we recognize that improvements to our model are possible, for example using random QTL effects, and that these may lead to even better results. Very recently, a fast analysis of all SNPs in a genome-wide association study was described using a method akin to a penalised likelihood approach [24]. This method was implemented to find a subset of SNPs that best explains case-control status in a disease study subject to a specified type-I error rate, but can also be used to select a subset for the prediction of phenotypes.

When comparing results between different prediction strategies, the accuracies of the intra-family prediction were generally higher than those for inter-family prediction (Table 2). There are three possible explanations for this observation. Firstly, the prediction of phenotype within families can use both linkage (family) and linkage disequilibrium (population) information for detected gene effects, whereas the prediction across families can exploit only LD in the population. Secondly, there may be polygenic effects which were not captured by the SNPs but these can be captured when using the phenotypes of close relatives. Thirdly, in the data set that we used, effects due to the common environment shared by littermates are confounded with genetic effects. Therefore, if there are such non-genetic effects that cause resemblance between relatives (in particular fullsibs), then these could be partially captured by the polygenic terms and even by SNP genotype effects. Importantly, such non-genetic common family effects do not affect the inter-family prediction. It was shown that the difference of the accuracy of prediction with and without polygenic terms based on pedigree information was large for the intra-family prediction whereas it was much smaller for the inter-family prediction for %CD8 and MCH (Table 2). This observation makes sense in that polygenic or common environmental effects can be informative for the prediction within families, but are not relevant for prediction across families. For coat colour, this pattern was not evident, presumably because the phenotypes are not affected by non-genetic family effects.

Given phenotype and pedigree data, narrow- or broad-sense heritability (*h^2^*) for the quantitative traits can be estimated in the classical genetic model [40]. However, since the data set used in this study consisted of full sib families with no replicates for maternal performance of dam, maternal environmental effects or family non-genetic effects may not be well-separated from genetic effects estimated in the classical model using pedigree information. Therefore, our estimate of heritability from the polygenic additive model may be biased upwards. We also tried to fit epistatic effects for pairs of SNP in addition to additive and dominance effects (see [23] for more detail on the method used). However, the model including epistasis did not improve the accuracy of prediction for any trait (results not shown). This was probably because the sample size was not sufficient to capture epistatic effects or, alternatively, because epistatic interactions do not contribute much to genetic variance in our data set [41].

We showed the strength of the multiple SNP method used in this study, compared to a set of SNPs obtained from the single SNP regression method, which is currently widely used in standard genome scans (Figures 4, 5 and 6). Compared to the multiple SNP method, the single SNP analyses generate more apparently significant SNPs but our results suggest that it would be much more difficult to determine the number and location of causal variants. Both methods can provide SNP sets to predict unobserved phenotypes. The accuracies of prediction using SNPs obtained from single SNP regression were generally lower than those with the multiple SNP method (Table 4). This was probably due to the fact that the choice of SNPs was not optimum. For example, selecting only one significant SNP in a region might ignore the possibility of having two QTL in a region, or alternately that multiple SNPs are required to explain the variance due to a single QTL. In contrast, the multiple SNP method used Bayesian model selection which tested all possible models with a proper acceptance ratio according to the appropriate posterior distribution.

**Table 4:** Correlation (SD^a^) and MSE^b^ (SD) between actual and predicted phenotypes for the single SNP method when varying threshold to select significant SNPs.

Method	Coat colour			%CD8			MCH		
	correlation	MSE	number of SNP^d^	correlation	MSE	number of SNP	correlation	MSE	number of SNP
Multiple SNPs^c^	0.87 (0.05)	0.02 (0.02)	15.3 (4.3)	0.58 (0.05)	0.03 (0.04)	12.8 (3.2)	0.36 (0.09)	0.09 (0.07)	14.1 (4.4)
Single SNPs^c^									
threshold of LR = 10.83	0.85 (0.03)	0.01 (0.01)	38.7 (6.0)	0.44 (0.06)	0.09 (0.17)	32.4 (4.9)	0.32 (0.07)	0.17 (0.15)	37.9 (5.6)
threshold of LR = 15.14	0.87 (0.02)	0.01 (0.01)	20.0 (2.9)	0.41 (0.06)	0.11 (0.19)	11.9 (3.2)	0.31 (0.10)	0.17 (0.12)	15.4 (4.2)
threshold of LR = 21.14	0.88 (0.02)	0.01 (0.01)	11.8 (2.1)	0.34 (0.08)	0.05 (0.08)	4.0 (1.7)	0.30 (0.08)	0.24 (0.16)	7.1 (2.5)
threshold of LR = 24.24	0.88 (0.02)	0.01 (0.01)	10.1 (2.2)	0.32 (0.08)	0.09 (0.05)	2.7 (1.0)	0.25 (0.11)	0.28 (0.12)	4.4 (1.8)

a Standard deviation over 10 replicates.

b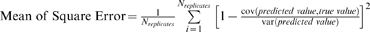.

c Inter-family prediction, using the **AD** model and fitting the pedigree.

d Number of SNPs contributing to predicting unobserved phenotypes for single SNP analysis, and averaged number of SNP to predicting unobserved phenotypes in each MCMC round for multiple SNP analysis.

We used a prior of Poisson distribution with mean *μ_n_* = 1 for the number of QTL (*nq*) in the RJMCMC. This might be a conservative way of detecting QTL, avoiding false positives and reducing random noise if there was no apparent prior information about the number of QTL. We also tested the performance of the RJMCMC with a different prior which was Poisson distribution with mean *μ_n_* = 14. Note that the estimated number of QTL was ∼15, ∼13 and ∼14 for coat colour, %CD8 and MCH, respectively (Table 4). Table 5 shows that the average number of SNP fitted simultaneously in each RJMCMC round was much larger with a prior mean of 14 than that with a prior mean of 1. However, the accuracy (correlation) was not much different whether using a prior mean of 1 or 14. Although the number of SNP simultaneously fitted in each RJMCMC round was smaller when using a prior mean of 1, all or most of the significant SNPs were found and fitted in the model over many iterations. This is why the accuracy with a prior mean of 1 is very close to that when using a prior mean of 14. This agrees with conclusions from previous studies [32],[34],[42] that estimation of QTL positions and effects are robust with respect to prior values. We also used a flat uniform prior for *ρ* (assuming that there was no prior information for the QTL positions), and ML estimates for *α* and *δ* were obtained given *nq* and *ρ* (also see online Supporting Information text S2). If there is apparent and useful information about priors, the RJMCMC can implement the information, which may give better results.

**Table 5:** Correlation (SD^a^) between actual and predicted phenotypes and the estimated number of contributing SNPs when using a prior from a Poisson distribution with a mean of 1 or 14.

Prior	coat colour		%CD8		MCH	
	correlation	number of SNP	correlation	number of SNP	correlation	number of SNP
mean = 1	0.87 (0.05)	15.3 (4.3)	0.58 (0.05)	12.8 (3.2)	0.36 (0.09)	14.1 (4.4)
mean = 14	0.88 (0.04)	36.9 (9.8)	0.56 (0.06)	35.7 (10.3)	0.35 (0.11)	35.2 (10.5)

Inter-family prediction, using the **AD** model and fitting the pedigree.

a Standard deviation over 10 replicates.

For our main RJMCMC analyses, we fixed the value of the polygenic heritability for computational reasons. We tested the sensitivity of this procedure on the accuracy of predicted phenotypes. For the polygenic heritability, three fixed values were compared, the previously used fixed value, half that value and a heritability of 0. In addition, we estimated heritability in every MCMC round. Table 6 shows that the accuracies are not dramatically different between estimates although zero heritability, equivalent to no polygenic effect fitted, results in slightly lower accuracies. We tested intra-family prediction only as this may be affected by value of the polygenic heritability.

**Table 6:** Fixed polygenic heritability used in the analyses and resulting correlation (SD^a^) between actual and predicted phenotypes.

coat colour		%CD8		MCH	
h^2^	correlation	h^2^	correlation	h^2^	correlation
0.72	0.89 (0.03)	0.99	0.73 (0.02)	0.55	0.55 (0.02)
0.36	0.91 (0.004)	0.50	0.70 (0.02)	0.28	0.54 (0.02)
0	0.85 (0.04)	0	0.69 (0.02)	0	0.5 (0.04)
unfixed	0.90 (0.09)	unfixed	0.71 (0.03)	unfixed	0.52 (0.02)

Intra-family prediction, using the **AD** model and fitting the pedigree.

a Standard deviation over 10 replicates.

Our results are based on ∼50% cross-validation. If more than 50% of the data are used for estimation then the accuracy of prediction may improve because estimates of marker effects will be more precise. We tested this by using 90% of the data for estimation stage and 10% for assessing the accuracy of prediction. Because families vary in size, it is not possible to select exactly 10% of each family. Therefore, we randomly divided the animals into 10 sets regardless of the family information. This generates a structure intermediate between inter- and intra-family prediction. We used 90% for estimation and 10% for validation and used 10 replicates without overlap in the validation sets. The correlation between true and predicted phenotypes and their SD were 0.91 (0.02), 0.73 (0.04) and 0.61 (0.06), for coat colour, %CD8 and MCH, respectively. The corresponding values for 50% cross-validation were 0.89 (0.03), 0.73 (0.02) and 0.55 (0.02). Hence, the accuracy for coat color and MCH are higher when using 10% cross-validation than when using 50% cross-validation, but the accuracy for CD8% is not much different. Standard deviation over 10 replicates tend to be larger with 10% cross-validation than that with 50% cross-validation. This is probably due to the fact that 90% discovery gives better estimation of marker effects and therefore we pick up a larger correlation, but that 10% validation gives larger sampling variance for the correlation.

Our MCMC method used estimated rather than sampled values for some parameters, which is known as an empirical Bayesian approach [43]. For a given QTL model, based on sampled values for the number of QTL, their effects and their positions, we obtained ML estimates for the remaining model parameters. This differs from the full Bayesian approach where in the MCMC algorithm all model parameters are sampled conditional on data and other parameters. Hence, the posterior distribution for the model parameters could differ somewhat from those of a full Bayesian approach. The empirical Bayesian approach has a large computational advantage as for sampled values for QTL number, effects and positions, no time is wasted with evaluating all possible values of Θ but rather evaluation is at the most likely value. Estimates converge more quickly compared to the full Bayesian approach. It is unlikely that much information is lost in this empirical Bayesian approach because parameters in Θ have smooth distributions and it is not likely that critical information exists at values with lower probability density. Casella [43] discussed the empirical Bayesian procedure for a hierarchical model where in an iterative procedure ML estimates were obtained for hyper parameters and other parameters were sampled conditional on these ML estimates. He justified this procedure statistically by showing that it implies an Expectation Maximization algorithm. In our approach, ML estimates for Θ and the likelihood of the data given the model parameters are used in RJMCMC to get the posterior density of QTL parameters across model dimensions. The justification for our procedure is shown in [20].

The method used here for prediction of phenotypes would be useful in many situations but the accuracy achieved is expected to vary. The mouse population was formed from crossbreeding inbred lines and so LD is expected to exist over considerable distance. In species with much less LD, for example humans, more markers and more phenotypic records are needed to achieve the same level of accuracy.

In conclusion, the prediction of unobserved phenotypes for complex traits from genome-wide marker data is feasible and can be accurate. Applications of our method are plentiful: in artificial selection programs it may lead to faster response to selection, by increasing the precision with which polygenic values are predicted [16], in human medicine it can be used to identify individuals that are most at risk for disease [17], and in forensics it can help to build a phenotypic profile from DNA evidence.

## Supporting Information

Text S1 RJMCMC Procedure.
(0.08 MB DOC)
