# Supplementary material for: Predicting Unobserved Phenotypes for Complex Traits from Whole-Genome SNP Data
Source: PLoS Genet. 2008 Oct 24;4(10):e1000231. doi: 10.1371/journal.pgen.1000231 (PMC2565502; doi:10.1371/journal.pgen.1000231)
Supplement: Text S1 — RJMCMC Procedure. (0.08 MB DOC) [file pgen.1000231.s001.doc]

SH Lee et al.

Supporting Information

Text S1: **RJMCMC procedure**

*RJMCMC probability for QTL birth*: For the move of the Markov chain across different model dimensions (the number of QTL is changed), a new QTL is proposed to be added (*nq*+1) with a proposal probability. A new QTL with position ($\rho_{n+1}$) is uniformly sampled from all unoccupied putative positions across the region. The proposal is accepted with probability,

$$a_{nq,nq+1}=\min\left\{1,\frac{pr(y|nq+1,\rho^{*},\Theta^{*})pr(nq+1)pr(\rho^{*}|nq+1)pr(\Theta^{*}|\rho^{*},nq+1)}{pr(y|nq,\rho,\Theta)pr(nq)pr(\rho|nq)pr(\Theta|\rho,nq)}\cdot\right.$$

$\left.\frac{pr(nq|nq+1)}{pr(nq+1|nq)}\cdot\frac{(nq+1)^{-1}pr(\Theta|\rho,nq)}{pr(\rho_{nq+1})pr(\Theta^{*}|\rho^{*},nq+1)}\cdot j\right\}$ (A1)

The first term in the right hand side is the posterior density consisting of the likelihood and the prior, and the second and third term are the proposal probability of adding or deleting a QTL from the model. The prior of the number of QTL (e.g. *pr*(*n*)) has a Poisson distribution with mean $\mu_{n}$=1. This is because there is no prior information available for the number of QTL, and this is a conservative way of detecting QTL, avoiding false positive. A flat uniform prior is used for *ρ,* and normal prior is used for *u*. ML estimates for *α* and *d* are obtained given *nq* and *ρ. pr*(*nq^*^|nq*) is a proposal probability of changing the number of QTL in the model from *nq* to *nq^*^* and *J* is the Jacobian of the transformation function probability from the current model to the other. Because adding or deleting a QTL in the method is the identity transformation, *J* is one ([1,2,3]). When deleting a QTL from the model, one QTL is randomly selected with a probability of (*nq*+1)^-1^, and the parameters involved in the selected QTL are removed from the model. ML estimates of the model parameters ($\Theta$) given the reduced number of QTL and their positions are determined with $pr(\Theta|\rho,nq)$. When adding a QTL to the model, $\rho_{nq+1}$ is used with its prior probability ([2]), and ML estimates of the model parameters given the increased number of QTL and positions are determined with $pr(\Theta^{*}|\rho^{*},nq+1)$. The equation can be rewritten as,

$a_{nq,nq+1}=\min\left\{1,\frac{pr(y|nq+1,\rho^{*},\Theta^{*})pr(nq+1)pr(\rho^{*}|nq+1)}{pr(y|nq,\rho,\Theta)pr(nq)pr(\rho|nq)}\cdot\frac{pr(nq|nq+1)}{pr(nq+1|nq)}\cdot\frac{(nq+1)^{-1}}{pr(\rho_{nq+1})}\cdot j\right\}$ (A2)

Following Jannink and Fernando (2004),

$\frac{pr(\rho^{*}|nq+1)}{pr(\rho|nq)pr(\rho_{nq+1})}=\frac{(nq+1)!\prod_{i=1}^{nq+1} pr(\rho_{i})}{nq!\left(\prod_{i=1}^{n} pr(\rho_{i})\right)\times pr(\rho_{nq+1})}=nq+1$ (A3)

This simplifies the acceptance ratio, $a_{nq,nq+1}$, as

$a_{nq,nq+1}=\min\left\{1,\frac{pr(y|nq+1,\rho^{*},\Theta^{*})}{pr(y|nq,\rho,\Theta)}\cdot\frac{pr(nq+1)pr(nq|nq+1)}{pr(nq)pr(nq+1|nq)}\cdot J\right\}$ (A4)

*RJMCMC probability for QTL death*: When deleting a QTL from the model, one QTL is randomly selected with a probability of *nq*^-1^, and the parameters involved in the selected QTL are removed from the model. ML estimates of the model parameters ($\Theta$) given the reduced number of QTL and their positions are determined with $pr(\Theta^{*}|\rho^{*},nq-1)$. The proposal is accepted with probability,

$$a_{nq,nq-1}=\min\left\{1,\frac{pr(y|nq-1,\rho^{*},\Theta^{*})pr(nq-1)pr(\rho^{*}|nq-1)pr(\Theta^{*}|\rho^{*},nq-1)}{pr(y|nq,\rho,\Theta)pr(nq)pr(\rho|nq)pr(\Theta|\rho,nq)}\cdot\right.$$

$\left.\frac{pr(nq|nq-1)}{pr(nq-1|nq)}\cdot\frac{pr(\rho_{nq})pr(\Theta|\rho,nq)}{nq^{-1}pr(\Theta^{*}|\rho^{*},nq-1)}\cdot j\right\}$ (A5)

The first term in the right hand side is the posterior density consisting of the likelihood and the prior, and the second and third term is the proposal probability of adding or deleting a QTL from the model. It is noted that when adding a QTL to the model, $\rho_{nq}$ is used with its prior probability ([2]), and ML estimates of the model parameters given the increased number of QTL and positions are determined with $pr(\Theta|\rho,nq)$. The equation can be simplified as,

$a_{nq,nq+1}=\min\left\{1,\frac{pr(y|nq-1,\rho^{*},\Theta^{*})pr(nq-1)pr(\rho^{*}|nq-1)}{pr(y|nq,\rho,\Theta)pr(nq)pr(\rho|nq)}\cdot\frac{pr(nq|nq-1)}{pr(nq-1|nq)}\cdot\frac{pr(\rho_{nq})}{nq^{-1}}\cdot j\right\}$ (A6)

Following Jannink and Fernando (2004), but modifying to suit QTL death,

$\frac{pr(\rho^{*}|nq-1)pr(\rho_{nq})}{pr(\rho|nq)}=\frac{(nq-1)!\left(\prod_{i=1}^{nq-1} pr(\rho_{i})\right)\times pr(\rho_{nq})}{(nq)!\left(\prod_{i=1}^{nq} pr(\rho_{i})\right)}=nq^{-1}$ (A7)

This simplifies the acceptance ratio, $a_{nq,nq-1}$, as

$a_{nq,nq-1}=\min\left\{1,\frac{pr(y|nq-1,\rho^{*},\Theta^{*})}{pr(y|nq,\rho,\Theta)}\cdot\frac{pr(nq-1)pr(nq|nq-1)}{pr(nq)pr(nq-1|nq)}\cdot J\right\}$ (A8)

Text S2: **Prior distribution of the number of additive and dominance effects**

In the **A** model, the number of additive effects is determined by the number of QTL, i.e. the number of additive effects (*ne*) = the number of QTL (*nq).* In the **AD** model, additive and dominance terms are entered or dropped together. Therefore, the number of effects is 2 times larger than the number of QTL, i.e. *ne* = 2 x *nq.* In a more formal way considering a prior of the number of effects, the acceptance ratio, $a_{nq,nq+1}$ (A4), can be written as,

$a_{nq,nq+1}=\min\left\{1,\frac{pr(y|nq+1,\rho^{*},\Theta^{*})}{pr(y|nq,\rho,\Theta)}\cdot\frac{pr(nq+1)pr(ne+1)pr(nq|nq+1)}{pr(nq)pr(ne)pr(nq+1|nq)}\cdot J\right\}$ (A9)

$a_{nq,nq+1}=\min\left\{1,\frac{pr(y|nq+1,\rho^{*},\Theta^{*})}{pr(y|nq,\rho,\Theta)}\cdot\frac{pr(nq+1)pr(ne+2)pr(nq|nq+1)}{pr(nq)pr(ne)pr(nq+1|nq)}\cdot J\right\}$ (A10)

Note that (A9) is for the **A** model, and (A10) is for the **AD** model. However, the prior information about the number of additive effects or dominance effects is unknown in these data. Therefore, we use $\frac{pr(ne+1)}{pr(ne)}$=1, and $\frac{pr(ne+2)}{pr(ne)}$=1, i.e. the number of the additive effects and dominance effects are estimated based on a flat uniform prior.

REFERENCES

1. Yi N, Xu S (2000) Bayesian Mapping of Quantitative Trait Loci under the identity-bydescent-based variance component model. Genetics 156: 411-422.

2. Jannink J-L, Fernando RL (2004) On the Metropolis-Hastings acceptance probability to add or drop a quantitative trait locus in Markov chain Monte Carlo-based Bayesian analyses. Genetics 166: 641-643.

3. Sillanpaa MJ, Arjas E (1998) Bayesian mapping of multiple quantitative trait loci from incomplete inbred line cross data. Genetics 148: 1373-1388.
